# Cross-species modeling of muscular dystrophy in *Caenorhabditis elegans* using patient-derived extracellular vesicles

**DOI:** 10.1242/dmm.050412

**Published:** 2024-04-02

**Authors:** Rewayd Shalash, Mor Levi-Ferber, Coral Cohen, Amir Dori, Chaya Brodie, Sivan Henis-Korenblit

**Affiliations:** ^1^ The Mina & Everard Goodman Faculty of Life Sciences, Bar-Ilan University, Ramat-Gan 52900, Israel; ^2^ The Mina and Everard Goodman Faculty of Life Sciences and Institute of Nanotechnology and Advanced Materials (BINA), Bar-Ilan University, Ramat-Gan 52900, Israel; ^3^ Department of Neurology, Sheba Medical Center, Ramat-Gan 52621, Israel; ^4^ Faculty of Medicine, Tel-Aviv University, Tel-Aviv 69978, Israel

**Keywords:** *Caenorhabditis elegans*, Duchenne muscular dystrophy, Disease modeling, Extracellular vesicles

## Abstract

Reliable disease models are critical for medicine advancement. Here, we established a versatile human disease model system using patient-derived extracellular vesicles (EVs), which transfer a pathology-inducing cargo from a patient to a recipient naïve model organism. As a proof of principle, we applied EVs from the serum of patients with muscular dystrophy to *Caenorhabditis elegans* and demonstrated their capability to induce a spectrum of muscle pathologies, including lifespan shortening and robust impairment of muscle organization and function. This demonstrates that patient-derived EVs can deliver disease-relevant pathologies between species and can be exploited for establishing novel and personalized models of human disease. Such models can potentially be used for disease diagnosis, prognosis, analyzing treatment responses, drug screening and identification of the disease-transmitting cargo of patient-derived EVs and their cellular targets. This system complements traditional genetic disease models and enables modeling of multifactorial diseases and of those not yet associated with specific genetic mutations.

## Teaser

**Summary:** Extracellular vesicles isolated from serum from patients with Duchenne or Becker muscular dystrophies can induce pathological processes in *Caenorhabditis elegans*, establishing a new approach for disease modeling in simple model organisms.

## INTRODUCTION

Animal models are essential for promoting biological and medical studies and allow analysis of molecular and pathophysiological pathways of diseases and pathological conditions. Such models enable repurposing of approved drugs and development of novel therapeutics. Rodents are the most common models of human diseases; however, studies using these models are hampered by species-related differences and ethical and budgetary concerns (Azkona and Sanchez-Pernaute, 2022; Jucker, 2010; Vandamme, 2014). Thus, alternative models, tightly related to human physiology and disease pathologies are required. Studies using simple model organisms, although distant from human physiology, are useful and practical to study evolutionary conserved processes.

Duchenne muscular dystrophy (DMD) and Becker muscular dystrophy (BMD) are the most common inherited muscle disorders, with DMD affecting one in 5000 and BMD affecting one in 15,000 live male births, with no effective cure (Crisafulli et al., 2020; Mendell et al., 2012). These dystrophinopathies are caused by mutations in the dystrophin gene (*DMD*) that can be either spontaneous or inherited (Bushby et al., 2010, 1993). Dystrophin is a subsarcolemmal protein expressed in skeletal, cardiac and smooth muscles and critical for muscle membrane integrity. It plays a critical role in the maintenance, integrity and normal functions of muscle cells, and its loss leads to progressive muscle degeneration and their replacement by fibrotic and fat tissues (Blake et al., 2002; Keefe and Kardon, 2015). Dystrophin is also essential for the asymmetric division of satellite cells and, therefore, its deficiency is implicated in the impaired muscle regeneration in patients (Dumont et al., 2015). The clinical manifestations of DMD include progressive muscle weakness and wasting, loss of ambulation by the age of 13 years and death at early adult age owing to cardiac or respiratory failure (Annexstad et al., 2014; Flanigan, 2014; Salmaninejad et al., 2018). Currently, the main treatment for DMD is glucocorticoids. Additional treatments are being developed for DMD (Duan et al., 2021), including anti-inflammatory and anti-fibrotic agents (Bushby et al., 2010; Heier et al., 2013; Huebner et al., 2008; Levi et al., 2015) and agents increasing muscle regeneration (Bogdanovich et al., 2002). A number of treatments that alter dystrophin expression have been recently approved for the treatment of DMD, including exon-skipping antisense oligonucleotides (Aartsma-Rus et al., 2017), such as Exondys51 and Viltepso, and the adeno-associated viral vector-based gene therapy, ELEVIDYS (Heydemann and Siemionow, 2023). Despite these efforts, there is currently no cure for these diseases.

Extracellular vesicles (EVs) are evolutionary conserved small membrane-bound vesicles, selectively loaded with cellular proteins, nucleic acid, lipids and additional molecules that can be transferred between cells. Due to packaging of their cargo in enclosed membranes, their content is stable and protected from degradation (Raposo and Stoorvogel, 2013; van Niel et al., 2018; Yáñez-Mó et al., 2015). EVs are secreted by a large variety of cells and circulate in biofluids, from which they can be extracted. Their content reflects the pathophysiological state of the originating cells. Hence, tissue and cell-derived EVs represent a viable and consistent reservoir of bioactive material that can be easily obtained from human fluids (Record et al., 2014; Riancho and Sánchez-Juan, 2018; van Niel et al., 2022). In the body, EVs interact with target cells and transfer their contents, which can then affect the functions of the recipient cells (Record et al., 2014). In this way, EVs play major roles in cell–cell communication in multiple physiological and pathological conditions. Accordingly, their role as circulating biomarkers and mediators of disease and pathological processes is currently being recognized (Mori et al., 2019; Stremersch et al., 2016; van Niel et al., 2022). EVs can also transfer cargo between organisms, either from the same or from different species. These abilities serve to evade the immune system of the recipient (Schorey et al., 2015), to affect brain vascular integrity (Xu et al., 2017) and to promote metastatic growth (Hyenne et al., 2019). Due to the intra- and inter-organismal roles of EVs, in-depth understanding of their functions as mediators of disease pathogenesis and propagation may have major clinical implications.

Here, we took advantage of the fact that EVs can cross inter-species barriers and analyzed the ability of EVs, isolated from the serum of patients with muscular dystrophies, to induce pathological processes associated with these diseases in naïve *Caenorhabditis elegans* recipients. We found that EVs isolated from different patients with BMD and DMD induced a similar robust disease-related muscle impairment in *C. elegans*, similar to that reported in genetic models of the disease. This demonstrates the potential use of patient-derived EVs to generate human disease models in simple model organisms, which can be used for analyzing the roles of EVs as mediators of disease pathological processes and for therapeutic screening.

## RESULTS

### EVs isolated from the serum of patients with DMD and BMD decrease *C. elegans* lifespan

To address the trans-species pathogenicity transduction potential by EVs derived from patients with BMD and DMD in the *in vivo* context of a whole animal, we isolated EVs from the serum of patients and applied them to a model organism that could potentially develop muscular dystrophy but does not carry genetic mutations in the dystrophin gene, which is the direct genetic cause of this disease. The model organism of choice was *C. elegans*, which is a widely used invertebrate model system for studying human diseases, including DMD, by standard genetic approaches (Ellwood et al., 2021a; Giugia et al., 1999; Grice et al., 2011; Hewitt et al., 2018; Oh and Kim, 2013). We isolated and characterized EVs from serum of two adult male patients with DMD, six adult male patients with BMD and four age- and gender-matched unaffected individuals. The average (±s.d.) age of the patients and unaffected individuals was 24.6±3.8 and 25.5±8.3 years, respectively. The patients with DMD and BMD differed in ambulation status and steroid treatment. However, except for two patients with BMD, the patients shared similarity in the status of cardiomyopathy. Likewise, except for two patients with BMD, the patients shared similarity in the Medical Research Council (MRC) score (Medical Research Council, 1943) of the right and left quadriceps. These reflect poor cardiac and limb muscle functions, respectively (Table S1).

All EV preparations were isolated, handled and stored under similar conditions. The EVs of the patients with muscular dystrophy and those from unaffected individuals exhibited similar characteristics as demonstrated by their average size and amount using nanoparticle tracing analysis (Fig. S1A), by CD81 and CD9 expression as demonstrated by western blot analysis (Fig. S1B), and by CD63 and CD81 expression using enzyme-linked immunosorbent assay (ELISA) (Fig. S1C).

**Fig. 1.**
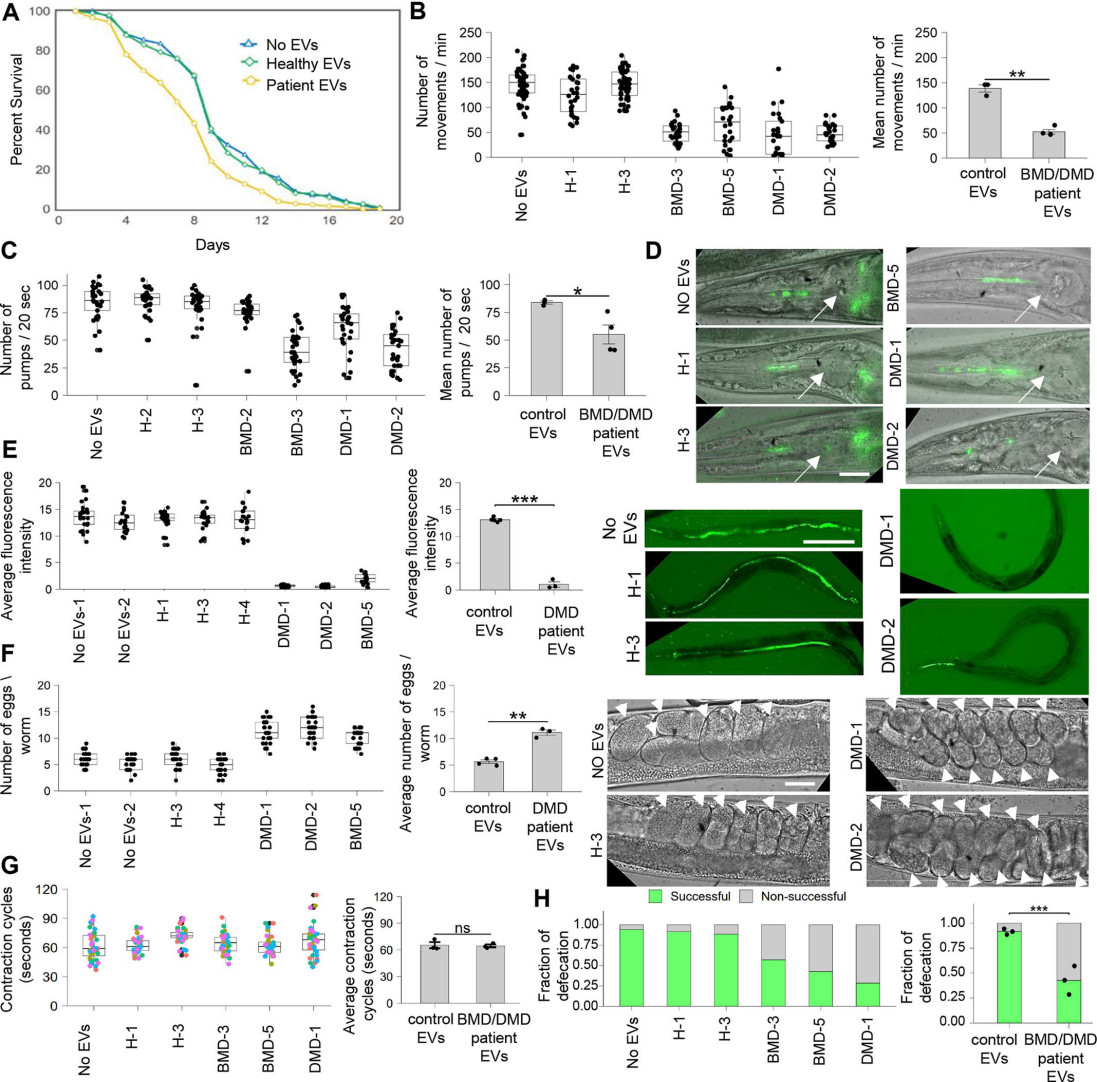
**EVs from patients with BMD/DMD impair *C. elegans* muscle functionality and decrease survival.** (A) Lifespan assay for *C. elegans* treated with no EVs (*N*=1, *n*=105), EVs from unaffected individuals (‘healthy EVs’) (*N*=2, *n*=195; *P*=0.902 compared to the ‘No EVs’ condition), EVs from patients with Duchenne muscular dystrophy (DMD) or Becker muscular dystrophy (BMD) (‘patient EVs’) (*N*=7, *n*=735; *P*=0.0000036). (B) Thrashing assay for *C. elegans* treated with control EVs (*N*=3, *n*=127) and patient EVs (*N*=4, *n*=103); *P*=0.001171. (C-E) Pharynx muscle functionality was assessed by pharynx contraction (pumps) (C) and by monitoring fluorescent food uptake past the grinder of the pharynx (D, arrows mark pharynx grinder) and into the intestine (E). (C) Control EVs (*N*=3, *n*=93), patient EVs (*N*=4, *n*=121); *P*=0.03898. (E) Control EVs (*N*=5, *n*=115), patient EVs (*N*=3, *n*=70); *P*=0.0004444. (F) Egg-laying defects. In-uterus embryos are marked by arrowheads. Control EVs (*N*=4, *n*=100), patient EVs (*N*=3, *n*=75); *P*=0.001265. (G) Defecation assays. The time between posterior intestinal contractions was scored for a total of 105 contractions. Control EVs (*N*=3, *n*=15), patient EVs (*N*=3, *n*=15); *P*=0.8386. (H) Defecation assays scoring the fraction of successful defecation events. Bars (left) represent proportions of successful defecation events in animals upon each EV treatment. Bars (Right) represent predicted proportions obtained from mixed-effects binary logistic regression of the control EV- or patient EV-treated animals. Control EVs (*N*=3, *n*=15), patient EVs (*N*=3, *n*=15). *P*<0.0001. Boxplots in B,C,E-G present the score distribution of individual animals upon each EV treatment. Each boxplot represents an independent biological replicate. Boxes show the interquartile range, whiskers are 1.5 times the interquartile range, and the median is marked with a line. The bar graphs for ‘control EVs’ average the mean scores of treatments with healthy EVs and control untreated animals and those for ‘BMD/DMD patient EVs’ average the mean scores of treatments with patient EVs, as indicated in the neighboring boxplots. *N* represents the number of treatment conditions, *n* represents the number of animals analyzed. Scale bars: 20 µm (D,F); 200 µm (E). ns, not significant; *P<0.05; **P<0.01; ***P<0.0001. Statistical tests: log-rank test (A); unpaired two-tailed Welch's two-sample *t*-test (B,C,E-G); binary logistic regression (H).

To test whether EVs derived from patients with DMD and BMD can transfer disease-inducing factors, *C. elegans* were raised from eggs to L4 in liquid growth medium supplemented with EVs derived from two unaffected individuals (‘healthy EVs’), seven patients with BMD/DMD (‘patient EVs’) or without EV supplementation (‘no EVs’). Young adult animals were transferred to standard growth plates and their viability was scored daily. We found a significant decline in the lifespan of animals treated with six different patient-derived EVs (EVs derived from the seventh patient also showed a lifespan shortening trend, albeit not statistically significant). Importantly, no lifespan shortening was observed upon treatment with EVs derived from age-and gender-matched unaffected individuals (Fig. 1A; Fig. S2), implying that the lifespan shortening activity was dependent on the source of the EVs and not a result of exposure to human EVs per se. These results demonstrate that patient-derived EVs can cross an interspecies barrier to affect *C. elegans* physiology and shorten their lifespan.

### DMD and BMD EVs decrease *C. elegans* muscle function

Patients with BMD/DMD are affected primarily by impaired muscle function and weakness. We examined whether EVs derived from patients with BMD/DMD compromise the functionality of different *C. elegans* muscles. In all cases, animals developed in the presence of human EVs until early adulthood and muscle functionality was assessed a few days later, as indicated per each experiment.

We first examined the effect of patient EVs on the body wall muscles of the animals, which are the functional equivalents of vertebrate skeletal muscles. Animal motility was assessed for day 5 animals in a thrashing swimming assay. Strikingly, whereas EVs derived from unaffected individuals did not significantly reduce animal motility in comparison to that of control untreated animals (no EVs), animals treated with patient EVs exhibited significantly decreased movement (Fig. 1B).

We next examined the effects of patient EVs on the functionality of *C. elegans* muscles related to food ingestion, reproduction and bowel movement. To assess food ingestion, the pumping rate of the nematode feeding organ, the pharynx, was scored. We found a reduction in the pharynx pumping rates in animals treated with EVs from three out of four patient donors compared with those in control untreated animals (Fig. 1C). Furthermore, when EV-treated animals were fed with a spike of fluorescent *Escherichia coli*, animals treated with patient EVs failed to pass the fluorescent food past the grinder of the pharynx (Fig. 1D). Accordingly, only residual amounts of fluorescent bacteria were detected in the intestines of these animals (Fig. 1E).

A functional reproductive system requires the coordinated activity of vulval and uterine muscles, as well as contractile gonadal sheath cells. Hence, impairment of these muscles is associated with egg-laying defects. Accordingly, deficits in egg-laying ability were observed in animals treated with EVs from patients with BMD/DMD. Specifically, this treatment resulted in accumulation of nearly 12 eggs per gonad, in contrast to animals treated with EVs from unaffected individuals that had approximately six eggs per gonad, similar to that for control untreated animals (Fig. 1F). This deficit in egg laying may directly reflect vulva muscle dysfunction but may also be an indirect effect of reduced food intake (Fig. 1C,D) (Trent et al., 1983).

Bowel movement in *C. elegans* requires the simultaneous contraction of four muscles near the anus: two intestinal muscles, the anal depressor and the anal sphincter. As periodical activation of a stereotyped sequence of these muscle contractions ends with defecation, their impairment is associated with constipation (Thomas, 1990). Consistent with impairment of bowel movement-related muscles, defecation deficits were observed in animals treated with EVs from patients with BMD/DMD. For scoring of defecation cycles, animals were fed with the fluorescent dye DiL, which is ingested by the animals and removed from the body as fluorescent defecation. We found that whereas defecation-associated muscle contraction intervals did not significantly change following treatment with patient EVs (Fig. 1G), only 25-62% of intestinal contractions led to successful defecation in animals treated with patient EVs (Fig. 1H). In contrast, nearly all intestinal contractions led to successful defecation in animals treated with healthy EVs as well as in untreated animals. Collectively, treatment with EVs from patients with BMD/DMD impaired all tested muscle functions in *C. elegans*, striated and non-striated muscles alike.

### DMD and BMD EVs induce disorganization of *C. elegans* muscle structure

**Fig. 2.**
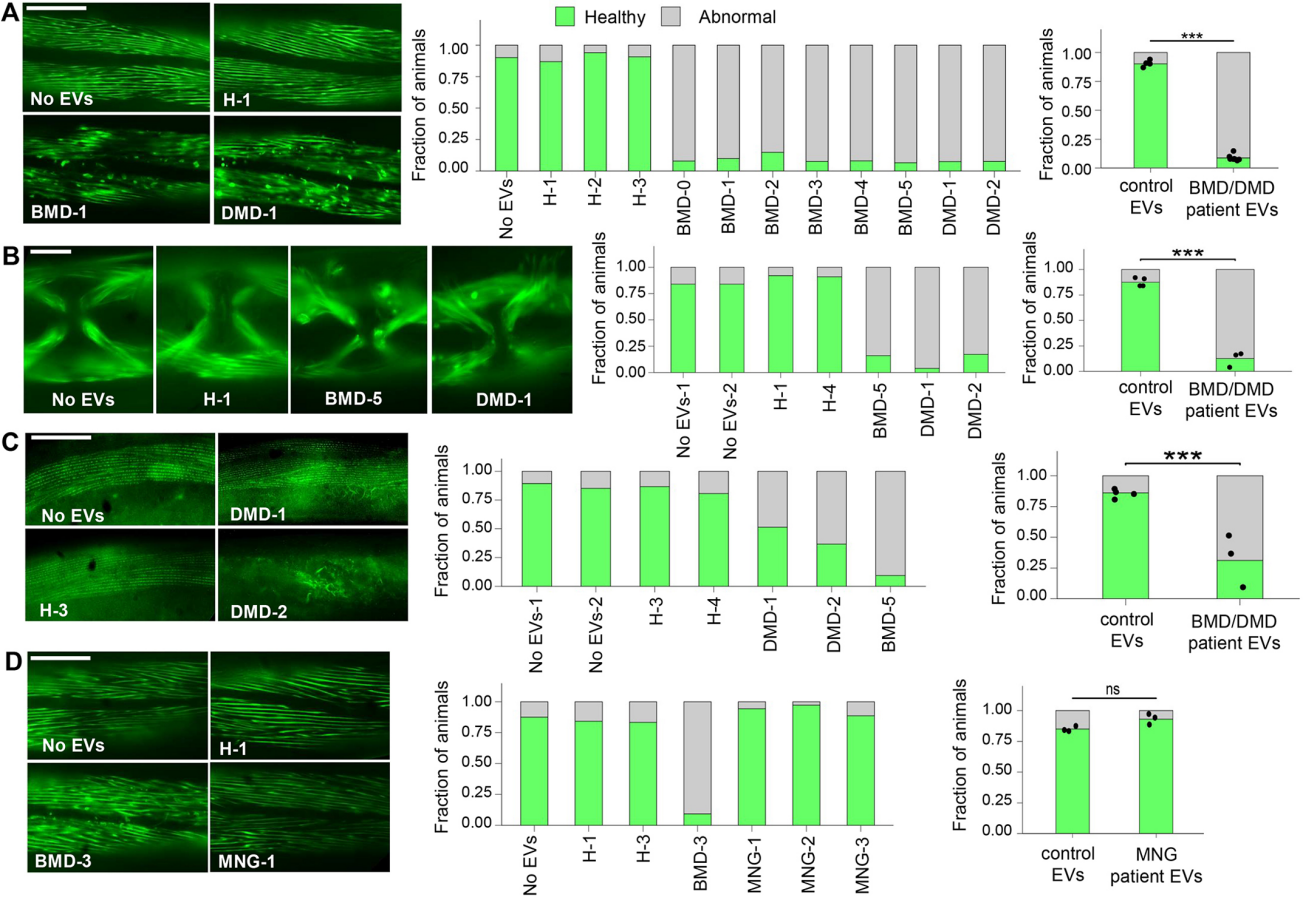
**EVs derived from patients with BMD/DMD impair *C. elegans* muscle structure.** (A-D) Body wall muscle myofilaments (A,D) and vulva muscle myofilaments (B) visualized by a MYO-3::GFP transgene, and body wall muscle *F*-actin filaments visualized by phalloidin staining (C). Scale bars: 100 µm (A,C,D); 20 µm (B). Bar graphs (left) represent proportions of healthy or abnormal muscle filaments upon each EV treatment, each bar representing an independent biological replicate. Bar graphs (right) represent predicted proportions obtained from mixed-effects binary logistic regression of the control EV (treatments with healthy EVs and control untreated animals, or patient EV-treated animals, as indicated in the neighboring bar graphs). (A) Control EVs (*N*=4, *n*=224), patient EVs (*N*=8, *n*=480); ***P<0.0001. (B) Control EVs (*N*=4, *n*=97), patient EVs (*N*=3, *n*=79); ***P<0.0001. (C) Control EVs (*N*=4, *n*=116), patient EVs (*N*=3, *n*=97); ***P<0.0001. (D) Control EVs (*N*=3, *n*=120), EVs from patients with meningioma (MNG) (*N*=3, *n*=115); ns, not significant, *P*<0.0546. *N* represents the number of treatment conditions, *n* represents the number of animals analyzed.

Impaired muscle function may be associated with muscle organization defects. To see whether muscle dysfunction upon treatment with EVs from patients with muscular dystrophy is also associated with irregular muscle structure, we analyzed myofilament organization in transgenic *C. elegans* expressing a MYO-3::GFP translational reporter driven by the *myo-3* promoter. This transgene produces a functional protein that localizes to myofilaments in vulva and body wall muscle cells (Meissner et al., 2009). We found that both vulva and body wall muscle myofilaments of animals treated with DMD/BMD EVs were disorganized and fragmented, whereas the myofilaments of animals treated with healthy EVs were well-organized and similar to those of non-treated animals (Fig. 2A,B; Fig. S3A). Strikingly, the myofilament disorganization phenotype was very robust and observed in nearly all body wall muscle cells in all animals treated with EVs from patients with BMD/DMD. Muscle organization defects were also observed upon phalloidin staining of actin filaments (Fig. 2C).

### EVs of patients with meningioma do not impair *C. elegans* muscle organization

We also assessed myofilament organization upon treatment of *C. elegans* with EVs isolated from patients with the brain tumor meningioma, which is not associated with muscle structure or function impairments. We found that the myosin fibers of animals treated with EVs from patients with meningioma were well organized, similar to those of non-treated animals (Fig. 2D; Fig. S3B). This suggests that the muscle-deleterious effects of human-derived EVs may be specifically associated with muscular dystrophy pathology.

### Prednisone protects *C. elegans* muscle from the deleterious effects of EVs from patients with DMD

**Fig. 3.**
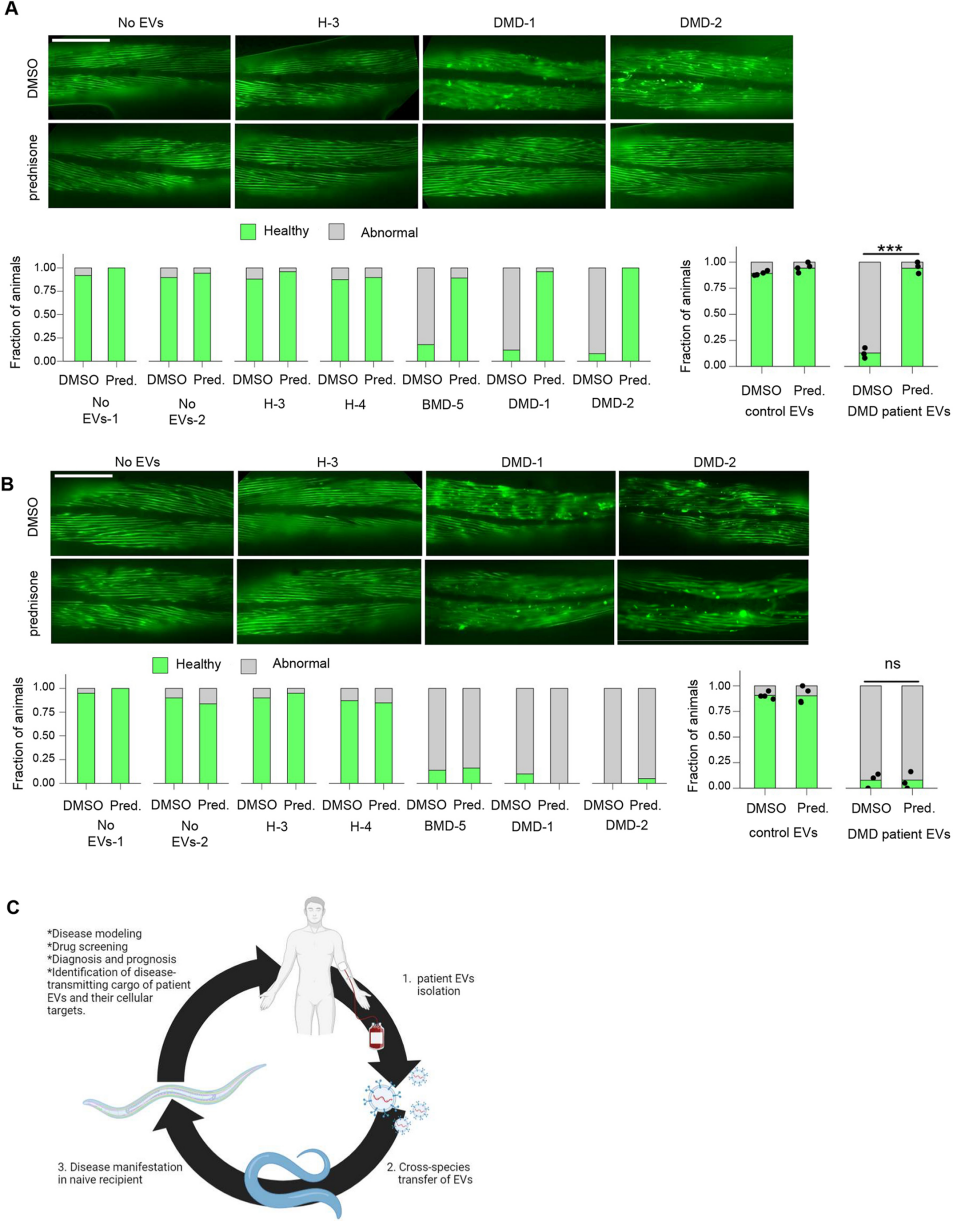
**Prednisone inhibits the effects of EVs derived from patients with BMD/DMD on the impairment of muscle structure in *C. elegans*, but does not resolve muscle atrophy.** (A,B) Body wall muscle myofilaments visualized by a MYO-3::GFP transgene in animals treated with DMSO or prednisone after incubation with control or patient EVs. Prednisone treatment was initiated either at L4 stage, prior to the first signs of muscle disorganization (A), or at day 2, after muscle disorganization had commenced (B). Scale bars: 100 µm. Bar graphs (left) represent proportions of healthy or abnormal muscle filaments upon each EV treatment, each bar representing an independent biological replicate. Bar graphs (right) represent predicted proportions obtained from mixed-effects binary logistic regression of the control EV- or patient EV-treated animals (as indicated in the neighboring bar graphs). (A) Control EVs DMSO (*N*=4, *n*=121), patient EVs DMSO (*N*=3, *n*=78), control EVs prednisone (*N*=4, *n*=125; *P*=0.1954 compared to the ‘control EVs DMSO’ condition), patient EVs prednisone (*N*=3, *n*=87; ***P=0.0001 compared to the ‘patient EVs DMSO’ condition). (B) Control EVs DMSO (*N*=4, *n*=109), patient EVs DMSO (*N*=3, *n*=69), control EVs prednisone (*N*=4, *n*=104; *P*=0.9772 compared to the ‘control EVs DMSO’ condition), patient EVs prednisone (*N*=3, *n*=77; ns, not significant, *P*=0.9772 compared to the ‘patient EVs DMSO’ condition). *N* represents the number of treatment conditions, *n* represents the number of animals analyzed. (C) Interspecies modeling of human diseases using patient-derived EVs. EVs isolated from patient serum can induce pathological processes associated with the disease in simple model organisms such as *C. elegans*, generating an interspecies disease modeling system that can be used for analyzing the roles of EVs as mediators of disease pathological processes and for therapeutic screening. Image created with BioRender.com.

The corticosteroid prednisone is the main current treatment for patients with DMD, particularly at an early age. We examined whether prednisone treatment delayed or reversed muscle disorganization induced by EVs from patients with BMD/DMD. To assess the therapeutic potential of prednisone treatment in the cross-species model system, *C. elegans* were raised until the L4 larval stage in the presence of EVs derived from serum of patients with DMD or unaffected individuals. At L4, animals were transferred to control plates or to plates supplemented with 0.37 mM prednisone, and myofilament structure was assessed in day 3 adult animals. Strikingly, the myofilaments of prednisone-treated animals remained organized and intact even in animals treated with patient EVs (Fig. 3A).

Next, we administered the prednisone treatment to *C. elegans* treated with EVs from patients with BMD/DMD at a later timepoint at which EV-induced muscle disorganization was already apparent (day 2 of adulthood). Myofilament structure was assessed in day 3 animals as before. Under these treatment conditions, the myofilaments of animals treated with prednisone from day 2 of adulthood were fragmented and disorganized, similar to those of animals not treated with prednisone (Fig. 3B). These results indicate that the timing represents a critical factor in its therapeutic effect in the context of muscle disorganization induced by EVs from patients with BMD/DMD in *C. elegans.*

## DISCUSSION

Reliable animal disease models that accurately recapitulate human disease pathologies are important for the understanding of pathophysiological processes, drug development and their translation into therapeutics for human diseases (Saeidnia et al., 2015; Sura et al., 2021). Studies using simple model organisms are useful and practical for studying evolutionary conserved processes, and hence may provide an accessible and reliable system for whole-animal disease modeling and for acceleration of translational and pre-clinical research (Apfeld and Alper, 2018; Hunter, 2023; Patten et al., 2016). Despite their genetic distance from humans, non-mammalian animal models such as *C. elegans*, *Drosophila* and zebrafish have been proven useful for the modeling of various human diseases (Baldridge et al., 2021; Boycott et al., 2020; Wangler et al., 2017). In most cases, human disease modeling involves the introduction of a pathology-associated mutation in a conserved gene or expression of a single human disease-associated transgene in the model organism (Capecchi, 2005). Generation of such models requires prior knowledge of the disease-causing mutation and they are best suited for modeling diseases associated with mutations in single genes (e.g. cystic fibrosis and sickle cell disease) (Botstein and Risch, 2003). However, many diseases and pathological conditions are polygenetic and multifactorial (e.g. heart disease, type 2 diabetes and aging) and, in other cases, the disease-associated mutations are unknown. All these generate a modeling challenge (Boyle et al., 2017). Another valuable approach to disease modeling relies on induced pluripotent stem cells from patient-derived cells, which can be potentially expanded and differentiated into any cell type (Brooks et al., 2022). However, studies of these models are limited to the cellular or organoid levels, and thus lack the whole organismal context of the disease. Here, we propose a previously unreported versatile inter-species platform to model human diseases in *C. elegans.* The platform takes advantage of patient-derived EVs, the contents of which reflect the complex pathophysiological state of the originating cells, combined with the natural abilities of EVs to transfer cargo between cells and organisms to mediate disease pathology and propagation.

As a proof of concept of the feasibility of this approach, which builds upon both EV pathogenicity and cross-species evolutionary conservation, we applied EVs from patients with muscular dystrophies to otherwise wild-type *C. elegans* and demonstrated their sufficiency to induce widespread muscle dystrophy-related pathologies (Fig. 3C). Strikingly, the EV-induced muscle dystrophy was robust and observed in nearly all animals exposed to the EVs, affecting the organization and function of both the striated-like body wall muscles and the pharynx, intestine, vulva and anus muscles. Interestingly, although DMD and BMD differ in the severity of disease manifestation, EVs derived from the serum of these patients exerted a similar pattern of pathological effects in *C. elegans*. This suggests that a common pathology-induced component in the EV cargo is present in all the EVs from patients with muscular dystrophy regardless of their disease status. The observed muscle deterioration upon treatment with patient EVs is reminiscent of muscle-related deficiencies typically seen for patients with muscle dystrophy. These changes reliably phenocopied muscle-deterioration phenotypes of the genetic models of DMD disease in *C. elegans*, induced by mutations in the dystrophin gene (Bessou et al., 1998; Ellwood et al., 2021b; Gieseler et al., 2000). These findings imply that muscle dystrophy-associated pathogenicity can be transferred, at least in part, by the patient EV cargo, with no obligatory requirement for genetic predisposition in the form of a mutation in the dystrophin gene in the recipient organism.

Although there is currently no cure for BMD/DMD diseases, prednisone is the most common approved treatment for patients, which can delay the loss of ambulation (Guglieri et al., 2022; Mackenzie et al., 2021). Interestingly, in our cross-species muscle dystrophy model system, treatment with prednisone inhibited muscle degeneration in animals treated with EVs from patients with BMD/DMD only when given prior to the manifestation of the disease symptoms in the worm. Consistent with this finding, it has been suggested that prednisone may affect the plasma membrane of muscle cells, leading to sarcolemma stabilization in the early steps of muscle degeneration (Brouilly et al., 2015; Gaud et al., 2004). Taken together, these findings suggest that prednisone treatment may exhibit higher efficacy for patients with BMD/DMD when administered prior to the early steps of muscle degeneration. These results emphasize the importance of this disease model for drug development and screening.

Importantly, muscle deterioration was not a common phenomenon in response to the treatment of *C. elegans* with any human EVs, as animals treated with EVs of unaffected individuals or patients with the brain tumor meningiomas maintained normal muscle organization and functions. This suggests that the pathogenicity propagation capacity of the EVs from patients with BMD/DMD may be disease-specific rather than a general consequence of exposure of the animals to human EVs. Such pathological cross-species effects may be mediated by a conserved target of the EV pathology-induced cargo in the recipient organism such as specific miRNAs and metabolites, which are partially conserved among species (Bartel, 2018; Liska et al., 2023).

We speculate that, similar to the ability of EVs to play a role in disease propagation in muscular dystrophy, patient-derived EVs may transfer disease-relevant components between species in additional pathological conditions. For this study, we obtained EVs from human blood, which contains a variety of tissue-derived EVs, representing a viable and consistent reservoir of bioactive material from all tissues that are in contact with the blood (Shah et al., 2018). Hence, as long as pathology-inducing cargo is present in the patient EVs, it can transfer the disease pathology between species, provided that the targets of this cargo are conserved as well.

Under these conditions, the cross-species disease modeling platform can be exploited to establish *ad hoc* personalized human disease models and provide an effective alternative to the use of mammalian models. This previously unreported modeling platform may be useful for diagnosis, prognosis, analyzing treatment responses, drug screening and identification of disease-transmitting cargo of EVs from patients and their cellular targets. This system complements traditional genetic disease models and provides an opportunity to model diseases that are not associated with specific genetic mutations or multifactorial diseases, and to identify disease propagation factors in the context of a whole organism. The approach presented here is simple (EVs can be obtained from patient biofluids), scalable (rapid generation of disease models in different genetic backgrounds) and personalized (EVs from different patients may be different), maximizes the efficient use of precious patient-derived material (owing to the microscopic size of *C. elegans*), and focuses on processes likely to be conserved between humans and the model organism (owing to the inter-species barrier and intra-species validation step). Altogether, this approach represents a unique, innovative and effective platform facilitating the study of disease induction, propagation and treatments, which can be further exploited for establishing a variety of novel personalized human disease models (Fig. 3C).

## MATERIALS AND METHODS

### Isolation of EVs from serum of patients and healthy volunteers

The Helsinki (Institutional Review Board) number for the EV study is 6525-19 and SMC 4813-17, granted by the Helsinki Committee of Sheba Medical Center, Ramat-Gan, Israel. EVs were isolated from serum samples obtained from patients with DMD, BMD and meningioma, and age- and gender-matched unaffected individuals using the ExoQuick PLUS kit (System Biosciences, Palo Alto, CA, USA) according to the manufacturer's instructions. The EVs were resuspended in sterile PBS and protein concentration was measured by the Lowry protein quantification method. EV samples were labeled per patient such that samples used for experiments were not anonymized. EVs were isolated, handled and stored under similar conditions. Following isolations, EVs were stored at −80°C in small aliquots to avoid freeze-thaw cycles. Informed consent was obtained from all human subjects.

### *C. elegans* strains

The following *C. elegans* strains were used: N2 [WB-STRAIN:N2_(ancestral)] and DM8005 {raIs5 [myo-3p::GFP::myo-3+rol-6(su1006)], RRID:WB-STRAIN:WBStrain00006128}.

### *C. elegans* treatment with serum-derived EVs

In all experiments, synchronized *C. elegans* eggs were allowed to develop until the L4 stage in 96-well plates at 25°C with gentle shaking. Each well contained 50 µl final volume of liquid growth medium composed of 15 µl dead OP50 (RRID:WB-STRAIN:OP50; killed by boiling for 40 min), 30 µl M9, 5 µl of 10 µg/ml EVs isolated from the serum of unaffected individuals, patients or with PBS as a control (no EVs). Synchronized L4 animals were transferred from the 96-well plates to standard NGM plates seeded with *Escherichia coli* OP50 and maintained at 25°C henceforth until scoring for lifespan, muscle function or muscle structure.

### Western blotting

EVs were resuspended in Mammalian Protein Extraction Reagent (M-PER, Thermo Fisher Scientific, Oregon City, OR, USA) supplemented with a protease inhibitor cocktail (Sigma-Aldrich, St. Louis, MO, USA). 20 µg of protein extracts were separated on 6-12% SDS gels, transferred onto polyvinylidene fluoride (PVDF) membranes and blocked. The membranes were probed with the primary antibodies [anti-CD9 (sc13118, Santa Cruz Biotechnology, Dallas, TX, USA; RRID:AB_627213; 1:200) and anti-CD81 (sc-166028, Santa Cruz Biotechnology; RRID:AB_2275895; 1:200)], washed and exposed to the secondary antibody [goat anti-mouse HRP-conjugated antibody (170-6516, Bio-Rad Laboratories, Hercules, CA, USA; RRID:AB_11125547; 1:10,000)]. The intensity of the bands was determined with the Immobilon Forte Western HRP substrate (Millipore, Burlington, MA, USA).

### CD63 and CD81 ELISA

The expression of CD63 and CD81 in isolated EVs was quantified using the ExoELISA-Ultra CD63 and CD81 kits (System Biosciences, Palo Alto, CA, USA) according to the manufacturer's instructions.

### Nanoparticle tracking analysis

Size distribution and concentration of the isolated EVs were measured using the NanoSight device (NS300, Malvern Instruments, MA, USA). EV samples were diluted in a 1:1000 ratio, and 1 ml of the diluted sample was loaded into the NanoSight device to monitor EV Brownian motion three times for 60 s. The captured live image was obtained from a screen gain of 6, camera level of 10 and detection threshold of 2. The results were analyzed using NanoSight particle tracking software (NTA 3.4). Particle concentration was determined by counting total particles and presenting the concentration as a histogram.

### *C. elegans* lifespan experiments

Wild-type eggs were treated with EVs until L4 stage as described above. Henceforth, aged-synchronized worms were raised on standard NGM media seeded with *E. coli* OP50 throughout life. Experimental groups contained 100 animals that were transferred to fresh plates every 1-2 days and scored for their survival. A worm that did not respond to three gentle touches on the head was considered as dead. Animals that ruptured or crawled off the plates were included as censored worms. SPSS software (IBM) was used to determine the means and *P*-values. *P*-values were calculated using the log-rank (i.e. Mantel–Cox) method.

### *C. elegans* thrashing assay

Wild-type eggs were treated with EVs until L4 stage as described above. Henceforth, synchronized animals were raised on NGM plates until day 5 of adulthood in 25°C. On day 5, the animals were placed in M9 buffer in 24-well plates and the frequency of lateral swimming movements was scored. Each animal was monitored for 20 s for thrashing after 10 s of habituation to the liquid. A movement of the worm that swung its head and tail to the same side was counted as one thrash. Values are presented as bends per minute.

### *C. elegans* pharyngeal pumping assay

Wild-type eggs were treated with EVs until L4 stage as described above. Henceforth, synchronized animals were raised on NGM plates until day 5 of adulthood in 25°C. Pharyngeal pumping of day 5 animals was assessed by counting the number of pharyngeal contractions during 20 s intervals.

### *C. elegans* food uptake assay

To follow bacterial intake, animals were fed with a GFP-expressing OP50 strain. Feeding experiments were performed by spreading 500 µl bacterial culture on the entire surface of a Petri dish. Day 3 animals were allowed to feed for 20 min and then washed twice with M9 to remove excess bacteria. The pattern of the fluorescent bacteria inside the animals was monitored under the microscope. Low-magnification imaging (100×) was used to defined three patterns of GFP within the animals: GFP fluorescence throughout the intestine (full), in some parts but not all of the intestine (partial) and lack of fluorescence in the intestine (none). High-magnification imaging (630×) was used to document bacterial fluorescence near the pharynx of the animals.

### *C. elegans* defecation assay

Wild-type eggs were treated with EVs until L4 stage as described above. Henceforth, synchronized L4 animals were raised on NGM plates until day 1 of adulthood at 25°C. To facilitate the scoring, animals were fed with the fluorescent dye Dil (Molecular Probes, D-282), which is ingested by the animals and removed from the body as fluorescent defecation, as previously described (Thomas, 1990). Briefly, day 1 animals were transferred and shaken in M9 solution containing 4 μg/ml DiL for 2 h. Then, animals were transferred to NGM plates and the total number of contractions anterior to the tails of the animals was scored over a 10 min period. Each contraction was scored as successful or failed based on the detection of fluorescent material exiting the animal upon each intestinal contraction.

### *C. elegans* egg laying

Wild-type eggs were treated with EVs until L4 stage as described above. Henceforth, late L4 animals were cultured on NGM plates for 36 h at 25°C. Animals were visualized and scored using Nomarski optics for eggs within their uterus.

### *C. elegans* fluorescence microscopy and quantification

To follow expression of fluorescent signals, animals were anaesthetized on 2% agarose pads containing 2 mM levamisole. Images were taken with a CCD digital camera using a Nikon 90i fluorescence microscope. For each trial, the exposure time was calibrated to minimize the number of saturated pixels and was kept constant through the experiment. NIS-Elements software (Nikon) was used to quantify mean fluorescence intensity as measured by intensity of each pixel in the selected area within the gonad.

### *C. elegans* phalloidin staining

Wild-type eggs were treated with EVs until L4 stage as described above. Henceforth, late L4 animals were cultured on NGM plates at 25°C until L4 stage as described above. Day 3 animals were washed in M9, frozen at −80°C, followed by a 5 min fixation in 100 µl pre-cooled methanol and a 5 min fixation in 100 µl pre-cooled acetone on ice. After removal of the acetone, animals were incubated in blocking solution (3% bovine serum albumin and 1% Tween 20 in PBS). After 20 min, the blocking solution was replaced with 100 µl of 750 µM rhodamine-phalloidin (Sigma-Aldrich, P5282) in blocking solution. Animals were stained for 1 h at room temperature in the dark under agitation. Staining was terminated by washes with the blocking solution.

### Statistical analysis

Data are presented as the mean values±s.e.m. and visualized in bar charts. For comparison between two data sets with continuous parameters (thrashing, pumping rate, *E. coli* fluorescence, egg laying and intestine contraction cycle assays), *P*-values were determined using unpaired two-tailed Welch's two-sample *t*-test. For lifespan experiments, *P*-values were calculated using the log rank (Mantel–Cox) analysis. For comparison between two data sets with non-continuous parameters (fraction of successful defecations, myofilament integrity and actin integrity assays), *P*-values were determined using binary logistic regression. Sample size estimates were not applied. Irresponsive and developmentally delayed animals were excluded from analysis. Animals were randomly selected for all experiments. Investigators were not masked to the group allocation during the experiment. Normality distribution was not tested due to the small sample size. Table S2 details all statistical data.

## Supplementary Material

10.1242/dmm.050412_sup1 Supplementary information

Table S2. Statistics
