# Supplementary material for: Cross-species modeling of muscular dystrophy in Caenorhabditis elegans using patient-derived extracellular vesicles
Source: Dis Model Mech. 2024 Apr 2;17(3):dmm050412. doi: 10.1242/dmm.050412 (PMC11007864; doi:10.1242/dmm.050412)
Supplement: Supplementary information [file dmm-17-050412-s1.pdf]

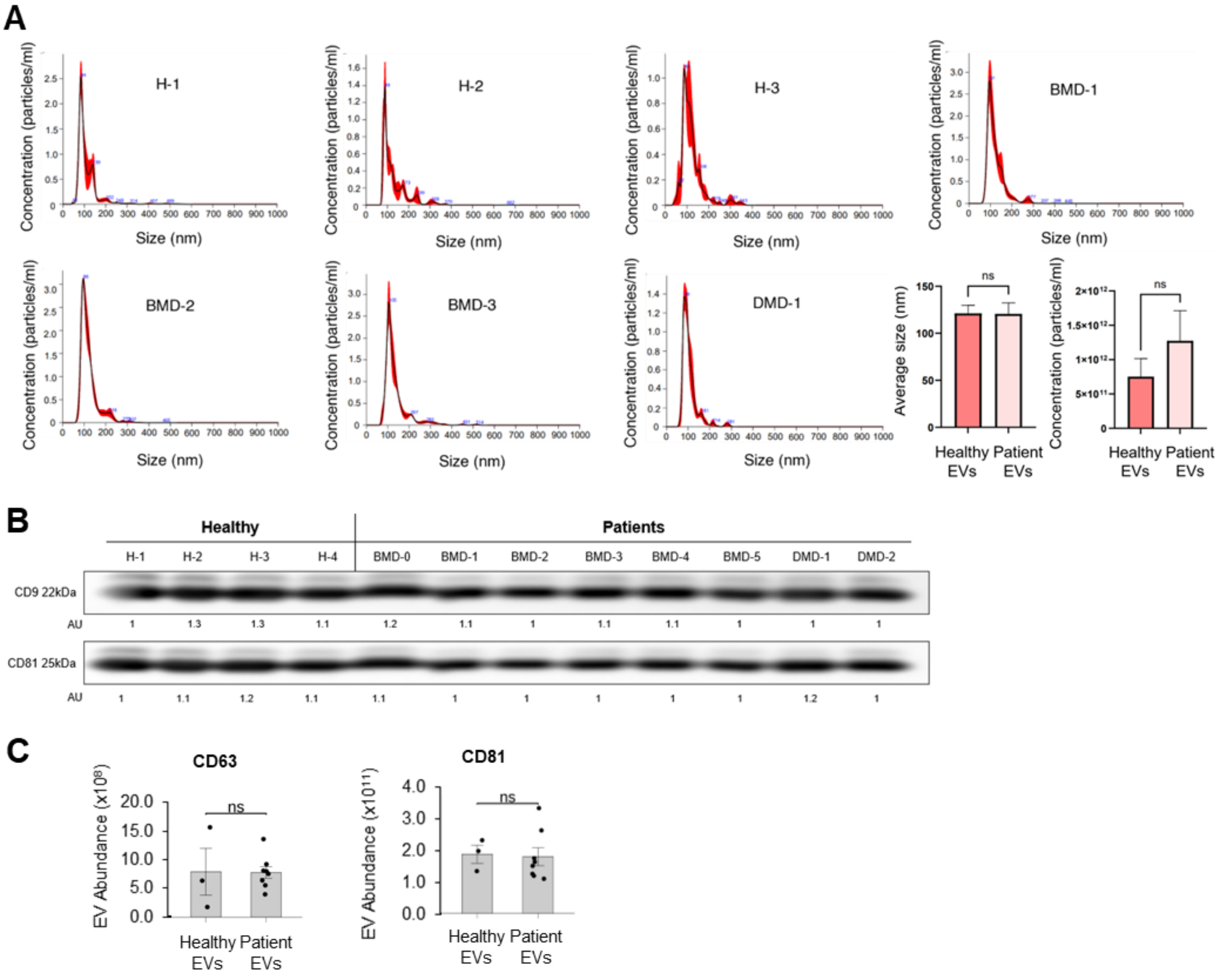

Fig. S1. Serum EV characterization. (A) EV concentrations and average size were determined by Nanoparticle Tracking Analysis (NTA) using NanoSight device (NS300). (B) CD9 and CD81 expressions were analyzed by Western blot. (C) Quantification of CD63+ and CD81 EVs was performed using ExoELISA-ULTRA kits. The bar graph represents the average expression of CD63 and CD81 in EVs isolated from healthy control (N=3) and DMD/BMD (N=8) serum EVs. Means ± SE values are presented. ns=not significant. P values were determined using unpaired Student's t-test.

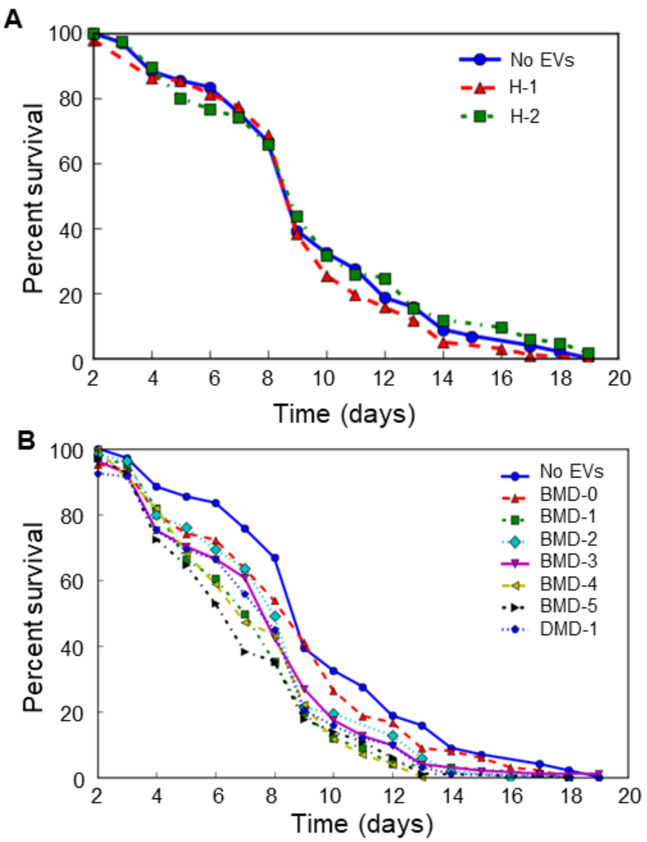

Fig. S2. BMD/DMD patient-derived EVs shorten *C. elegans* lifespan. Wild-type *C. elegans* animals were incubated with EVs derived from human serum from eggs till early adulthood and survival was scored thereafter. **(A)** Survival of animals incubated with EVs derived from healthy individuals (N=2) compared to animals not exposed to any human EVs (No EVs, N=1). **(B)** Survival of animals incubated with patient EVs (N=7) compared to animals not exposed to any human EVs (No EVs, N=1). Both graphs represent the same experiment and share the no EVs data. N represents independent biological replicates.105 animals were scored per each EV treatment.

Disease Models & Mechanisms • Supplementary information

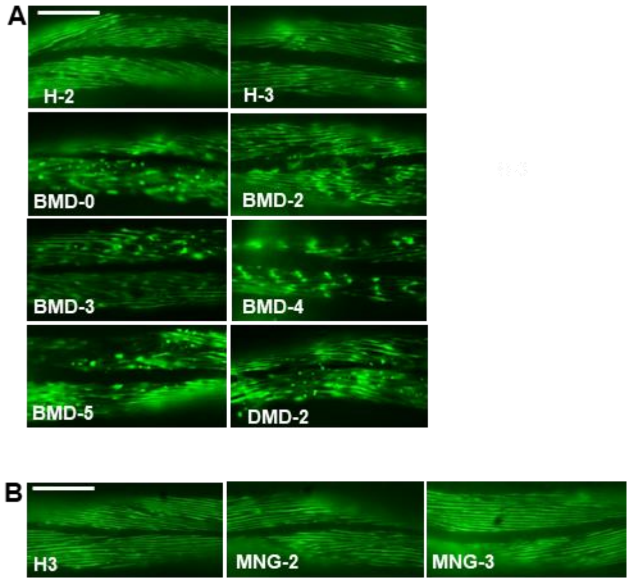

Fig. S3. BMD/DMD patient EVs impair *C. elegans* muscle structure, but MNG patient EVs do not. Representative images of body-wall muscles myofilaments visualized by a MYO-3::GFP transgene, in animals incubated with EVs derived from Healthy individuals (H-2, H-3), BMD/DMD patients (N=6) or MNG patients (N=2). Treatment with BMD/DMD patients EVs impaired myofilament organization **(A)** whereas animals treated with MNG patients EVs exhibited well-organized body wall muscles **(B)** similar to animals treated with EVs from healthy individuals. Scale bar 100µm.

Disease Models & Mechanisms • Supplementary information

Table S1. De-identified DMD and BMD patient information

	Sample	Sex	Age at sampling	Steroid treatment	Ambulation	MRC sum score	MRC score for QR and QL	Cardio-myopathy
Healthy	H-1	M	37	No	Yes	60	5	No
	H-2	M	21	No	Yes	60	5	No
	H-3	M	18	No	Yes	60	5	No
	H-4	M	26	No	Yes	60	5	No
BMD	BMD-0	M	25	No	Yes	60	5	No
	BMD-1	M	25	Yes	No	37	2	No
	BMD-2	M	25	Yes	No	30	2	Yes
	BMD-3	M	25	No	Yes	46	2	Yes
	BMD-4	M	22	No	Yes	54	4	Yes
	BMD-5	M	21	Yes	Yes	42	2	Yes
DMD	DMD-1	M	21	Yes	No	20	1	Yes
	DMD-2	M	33	Yes	No	4	0	Yes
Meningioma (Grade 1)	MNG-1	M	31	No	Yes	60	5	No
	MNG-2	M	36	No	Yes	60	5	No
	MNG-3	M	25	No	Yes	60	5	No

For each patient, age, steroid treatment, ambulation, and MRC scores are presented.

Table S2. Statistics

Available for download at
https://journals.biologists.com/dmm/article-lookup/doi/10.1242/dmm.050412#supplementary-data

Disease Models & Mechanisms • Supplementary information
